# Roles of histone hypoacetylation in LAT expression on T cells and Th2 polarization in allergic asthma

**DOI:** 10.1186/1479-5876-11-26

**Published:** 2013-01-30

**Authors:** Cheng-ye Li, Juan Peng, Lian-pin Ren, Li-xing Gan, Xiao-jiong Lu, Qian Liu, Wen Gu, Xue-jun Guo

**Affiliations:** 1 Department of Respiratory Medicine, Xinhua Hospital, Shanghai Jiao Tong University School of Medicine, 200092, Shanghai, China; 2 Division of Trauma, Surgical Critical Care and Burns, Medical Center of University of California San Diego, 92103, San Diego, CA, USA; 3 Present address: Department of Respiratory Medicine, The First Affiliated Hospital of Wenzhou Medical College, 325000, Wenzhou, China

**Keywords:** Linker for activation of T cells, Histone, Deacetylase, Acetylation, Methylation, Th2 cells, Allergic asthma

## Abstract

**Background:**

Linker for activation of T cells (LAT), a transmembrane adaptor protein, plays a role in T cell and mast cell function, while it remains unclear how histone modifications mediate LAT expression in allergic asthma. The present study aimed at understanding alterations of lymphocyte LAT in patients with asthma and potential mechanisms by which histone modulation may be involved in.

**Method:**

The expression of LAT mRNA was checked by Quantitative real-time PCR and histone hypoacetylation on LAT promoter was detected by Chromatin Immunoprecipitation.

**Results:**

Our results demonstrated that the expression of LAT mRNA in peripheral blood T cells from patients with asthma decreased, as compared to healthy controls. Peripheral blood T cells were treated with pCMV-myc-LAT, pCMV-myc or LAT-siRNA plasmid. Over-expression of LAT mRNA and decrease of Th2 cytokine production were noted, which could be prevented by the inhibition of LAT. The further investigation of the role of histone was performed in an asthma model induced by allergen. Histone hypoacetylation on LAT promoter could inhibit LAT expression and enhanced Th2 differentiation, while trichostatin A, a histone deacetylase inhibitor, promoted LAT expression and inhibited Th2 cytokine production.

**Conclusion:**

Our results indicate that histone hypoacetylation may regulate LAT expression on T cells and modify Th2 polarization in allergic asthma*.*

## Introduction

Linker for activation of T cells (LAT) is a transmembrane protein, which is expressed in thymocytes, mature T cells, NK cells, mast cells, megakaryocytes, and pre-B cells [1-4]. As lacking any intrinsic enzymatic activity, the ability of LAT to transmit signals depends upon its phosphorylation. Upon TCR engagement, phosphorylation of LAT allows it to interact with several SH2 domain-containing proteins, such as Grb2 and PLC-γ1. LAT serves to nucleate a large signaling complex upon TCR engagement that is essential for T-cell development and function [5]. It has been shown that mice homozygous for a single tyrosine mutation in LAT exhibited an early block in T cell maturation but later developed a polyclonal lymphoproliferative disorder that produced high amounts of Th2 cytokines. This exaggerated Th2 differentiation caused tissue eosinophilia and massive maturation of plasma cells secreting to immunoglobulins of the E and G1 isotypes [6,7]. These data suggest that LAT is crucial in regulating Th2 polarization.

Allergic asthma is a common health problem, which is characterized by chronic airway inflammation associated with a T helper (Th) 2 immune response to environment allergens [8,9]. However, whether LAT is involved in the pathogenesis of allergic asthma has not been clearly identified.

Modifications of core histones, including acetylation, methylation, phosphorylation and ubiquitination, play a critical role in alteration of chromatin structure and gene transcription [10]. Among those modifications, acetylation and methylation of histone lysine residue have been well characterized [11]. Histone hyperacetylation is generally associated with chromatin decondensation, allowing transcription activators access to DNA and increasing transcriptional activity, whereas histone hypoacetylation is linked with chromatin condensation and transcriptional repression [12,13]. It has been shown that the acetylation of histones is regulated by histone deacetylases (HDACs) [14]. Moreover, lysine methylation can exist in three different states, monomethylated (me1), dimethylated (me2), and trimethylated (me3). Generally, transcriptionally silent regions contain H3K9me3 (trimethyl), H3K27me2/3 (di- and trimethyl), and H4K20me1 (monomethyl), whereas active genes correlate with H3K4me2/3 (di- and trimethyl), H3K36me2/3 (di- and trimethyl), and H3K79me2 (dimethyl) [15-17]. However, the function of histone modification in LAT expression in allergic asthma has not been addressed.

In this study, we found that LAT expression was significantly decreased in T cells from asthmatic patients. Overexpression of LAT in T cells resulted in impaired Th2 polarization, whereas LAT deficiency enhanced Th2 cell development. In an allergen-induced asthma model, histone hypoacetylation on LAT promoter inhibited LAT expression and enhanced Th2 differentiation. In contrast, Trichostatin A (TSA), a histone deacetylase inhibitor, promoted LAT expression and attenuated Th2 cytokine production. Our results thus indicate that LAT, regulated by histone modification, is an essential regulator in Th2 polarization in allergic asthma.

## Materials and methods

### Human study subjects

Study participants were randomly selected from outpatients and inpatients of Xinhua Hospital, Shanghai Jiao Tong University. Twenty participants with allergic asthma (10 women, 10 men) and twenty participants (10 women, 10 men) were healthy controls. Asthma was diagnosed from common symptoms and pulmonary functions [18]. The severity of asthma was evaluated on the basis of the Global Initiative for Asthma criteria [19], both mild and moderate asthmatics were enrolled. The skin-prick test (SPT) was performed using a standard panel of 11 allergens, a 15 mm diameter skin wheal response was defined as positive. In the four weeks prior to the study, asthmatic participants were permitted to be treated with inhaled glucocorticoid but not systemic steroids.

The healthy control subjects with age and sex matched to allergic asthma participants were reported no allergic diseases, were negative SPT and had not received oral or intravenous steroids in the previous 4 weeks before the study.

All subjects were fully informed about the purpose and nature of the studies, which were approved by the medical ethics committee of Xinhua Hospital. Written informed consent was obtained from the patients for publication of this report and any accompanying images.

### Peripheral blood T lymphocytes

#### Isolation and purification

Twenty milliliters of fresh human peripheral blood was obtained from allergic asthmatics and healthy controls. Peripheral blood mononuclear cells (PBMCs) were isolated by density gradient centrifugation using Lymphoprep (d = 1.077 mg/ml; Nycomed Pharma AS, Roskide, Denmark). The purified blood T cells were subsequently obtained using T Cell Negative Isolation Kit (Invitrogen Dynal AS, Oslo, Norway) according to the manufacturer’s instruction. The purity of T cells was 90-95%.

#### Nucleofection

The purified peripheral blood T cells were nucleofected with pCMV-myc-LAT, pCMV-myc or LAT-siRNA plasmids (nucleofect with three kinds of plasmids in healthy controls and pCMV-myc-LAT, pCMV-myc plasmids in asthma patients) using the Human T Cell Nucleofector® Kit (Amaxa, Lonza, Germany). The protocol was done according to the manufacturer’s instruction.

#### Activation

The nucleofected T cells were activated with anti-CD3 (1 μg/ml, BD Bioscience) and anti-CD28 (1 μg/ml, BD Bioscience) and cultured for 24 h at 37°C with 5% CO_2_. Cells were harvested for Western Blot analysis and the supernatants were collected and preserved at −20°C for ELISA.

### Animal study

Male, 6-8-week-old Wistar rats from Sinobritish Sipprbk Lab Animal Ltd (Shanghai, China) were maintained under pathogen-free conditions at the animal center of Xinhua Hospital, Shanghai and were age- and sex-matched within each experiment. Mice studies were approved by the Xinhua Hospital Animal Care and Use Committee.

### Sensitization and airway challenge

Wistar rats were sensitized twice, with an interval of 7 days, by the intraperitoneal (i.p) injection of 0.5 ml of 2 mg chicken ovalbumin (OVA) (Grade V, Sigma-Aldrich, shanghai, china) bound to 200 mg aluminum hydroxide Al(OH)_3_ (Sigma) in saline. Simultaneously, 6 × 10^9^ heat-killed Bordetella pertussis bacilli were administered intraperitoneally as an adjuvant. From day 15 to day 28, rats were exposed to aerosolized OVA (1% in saline) or saline alone (Control groups) for 20 minutes once a day. The aerosol was generated with a nebulizer (PARIBOY N037, PARI, Starnberg, Germany) and was drawn into the exposure chamber containing the awake animals. Rats were euthanized (50 mg/kg pentobarbital, i.p.) 24 h after the final challenge, serum, bronchoalveolar lavage fluid (BALF) and lungs were sampled.

### Lung T cells isolation and activation with or without TSA

Lung lymphocytes were acquired by Lymphocytes Separation Medium (HISTOPAQUE 1083, Sigma, St. Louis, MO, USA). T lymphocytes were purified from mononuclear cells by nylon wool filtration. The purity of T cells was > 85%, as assessed by flow cytometry with anti-CD3-FITC antibody (eBioscience, San Diego, CA, USA). The purified lung T cells were incubated with or without TSA (2.5 ng/ml) for 24 h. After incubation, the supernatants were collected, pooled per stimulation for cytokine production analysis, and cells were harvested for either total protein and RNA isolation or ChIP assay.

### Cytokine measurement

The level of rats IL-4 and IFN-γ in BALFs and serum of immunized and non-immunized rats, and in the supernatants of cultured lung T cells with or without TSA were measured and quantified by ELISA following the manufacturer’s instructions (ELISA kit, invitrogen). As well as the production of IL-4 and IFN-γ in the human peripheral blood nucleofected T cells from allergic asthmatics and healthy controls.

### Quantitative real-time PCR

Total RNA in human peripheral blood T cells and rat lung T cells was extracted using TRIzol reagent (invitrogen) and 1 μg of RNA was reversed transcripted using cDNA synthesis kit (TaKaRa, Dalian, China). The primer specific for LAT (forward, 5’-GAGGATGTGGATGGAGAGGA-3’; reverse, 5’-CTGTAGGCA AGGCAGAGGTC-3’, which produced a 143 bp product) and GAPDH (forward, 5’-GCAAGTTCAACGGCACAG-3’; reverse, 5’-GCCAGTAGACTCCACGACAT-3’, which produced a 140 bp product), were designed and synthesized by Beyotime Institute of Biotechnology, Shanghai, China.

The quantitative real-time PCR was performed on an ABI PRISM 7500 Fast Real-Time PCR System (ABI, USA), and SYBR Green (TaKaRa, Dalian, China) was used as a double-stranded DNA-specific fluorescent dye. GAPDH was used as a housekeeping gene for standardizing LAT mRNA expression. After normalization of the data according to the expression of β-actin mRNA, relative levels of LAT and GAPDH were calculated using the 2^—△△Ct^ method [20].

### Western immunoblotting

Lung T cell and human nucleofected T cell protein extracts were prepared. Approximately 20–30 μg of protein was subjected to 10% SDS/PAGE gels and transferred to polyvinylidene difluoride membranes (Millipore Corporation, Billerica, MA, USA). After transfer, the membranes were blocked by 5% fat-free milk in TBST (20 mM Tris–HCl pH 7.6, 137 mM NaCl, 0.1% Tween-20) for 1 h at room temperature (RT) and incubated with primary antibodies against LAT (1:750) (Cell Signal), HDAC1 (1:750) (Cell Signal, Danvers, MA, USA), acetyl-H3 (1:20000) (Millipore Corporation), acety-H4 (1:2000) (Millipore Corporation) or dimethyl-H3K9 (1:500) (Millipore Corporation) overnight at 4°C in the TBST, and then incubated with an HRP-labeled secondary antibody (1:1000) (Beyotime Institute of Biotechnology, Shanghai, china) for 2 h at RT. Incubation with anti-GAPDH (1:1500) (Abcam, San Francisco, CA 94105, USA) served as a loading control. The blots were visualized by chemiluminescence with ECL Western blotting detection reagents (Millipore Corporation).

### ChIP Assay

The ChIP assay was carried out using a ChIP assay kit (Millipore) according to the manufacturer’s protocol with minor modification. Cultured lung T cells (~8 × 10^6^) were cross-linked by 0.4% formaldehyde at 37°C for 5 min, and then excess formaldehyde was quenched by addition of glycine at a final concentration of 0.125 M. Sonication was performed on ice using a Bioruptor sonicator to shear the cross-linked DNA to an average length of 200–1000 bp. Supernatants of the samples were collected and diluted 10 fold in ChIP dilution buffer (20 μl of each was reserved as total input control) followed with preimmunoprecipitation clearing with 80 μl of a Protein A Agarose/Salmon Sperm DNA −50% Slurry for 30 minutes at 4°C with agitation. Immunoprecipitation was performed with 4 μl of anti-acetylhistone H3, anti-acetylhistone H4 anti-acetylhistone H3 or anti-dimethylhistone H3 (K9) and incubated overnight at 4°C with rotation. Immune complexes were collected with 60 μl protein A agarose/salmon sperm DNA for 60 min at 4°C with rotation and washed twice with low salt buffer, once with high salt buffer, once with LiCl buffer, and twice with TE Buffer. The immune complexes were eluted twice from the antibody by with 250 ul elution buffer. The elutes and the input were heated at 65°C for 6 h to reverse histone-DNA croosslinks by the addition of 20 μl of 5 M NaCl. DNA was extracted using a DNA Mini Preparation Kit (Beyotime Institute of Biotechnology, Shanghai, china). 2 μl of each of the purified DNA was used as template for 32 cycles of PCR amplification. The PCR products were analyzed on 1.5% agarose gel. The following primers of rat LAT gene, covering the 5’-flanking region (−75/+23), were used: 5’-TGAGGAGCCTGATGATTTCC-3’; 5’-GCTG TACCTGCCTTTCTTGC-3’. Non-specific IgG (Beyotime Institute of Biotechnology, Shanghai, China) was served as the negative control in the assay.

### Statistical analysis

All data were expressed as mean ± SD. Differences between groups were calculated for statistical significance using the Student’s T-test. A P-value ≤ 0.05 was considered as statistically significant.

## Results

### LAT mRNA was decreased in peripheral blood T cells from allergic asthmatic patients

**Figure 1:**
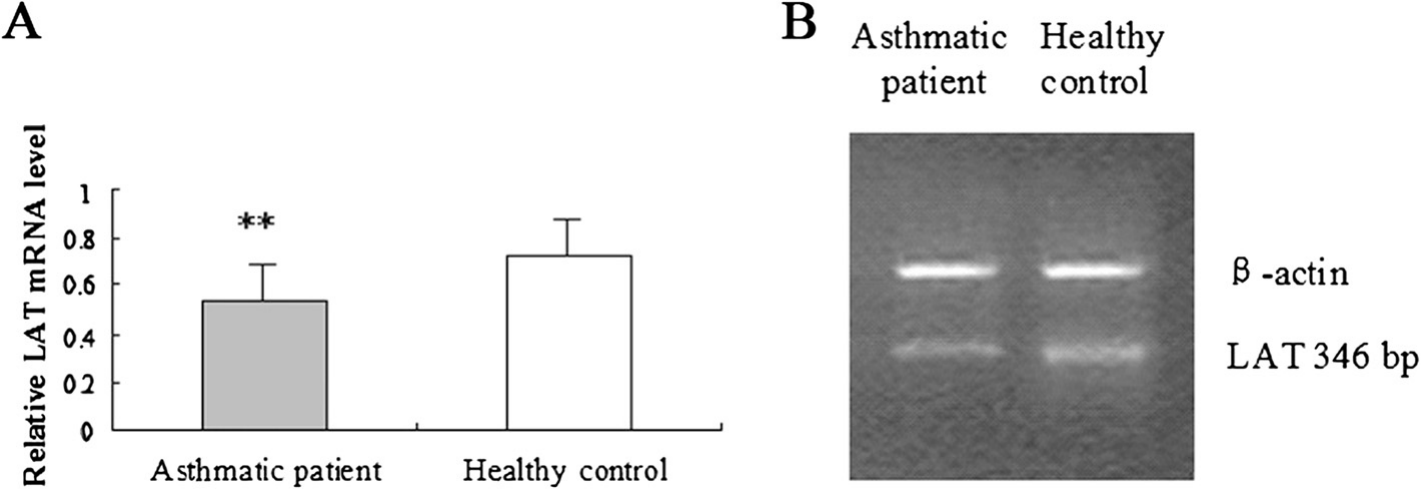
**Expression of LAT mRNA in peripheral blood T cells in asthmatic patients and healthy controls. A**) The mRNA expression of LAT was determined by real-time PCR in peripheral blood T cells from asthmatic patients and healthy controls, which was normalized to β-actin. The ratio of LAT/β-actin was determined as the relative content of LAT mRNA. Data is shown as mean ± SD, n = 10 (*p < 0.05). **B**) Image of RT-PCR analysis of LAT expression in asthmatic patients and healthy controls.

To determine the role of LAT in allergic asthma, the LAT mRNA expression on peripheral blood T cells from allergic asthmatic patients and healthy controls was evaluated. The relative LAT mRNA level was dramatically decreased in the peripheral blood T cells from allergic asthmatic patients compared to healthy controls (Figure 1A-B, *P* < 0.01), emphasizing the role of LAT in allergic asthma.

### LAT regulates the differentiation of peripheral blood T cells *in vitro*

**Figure 2:**
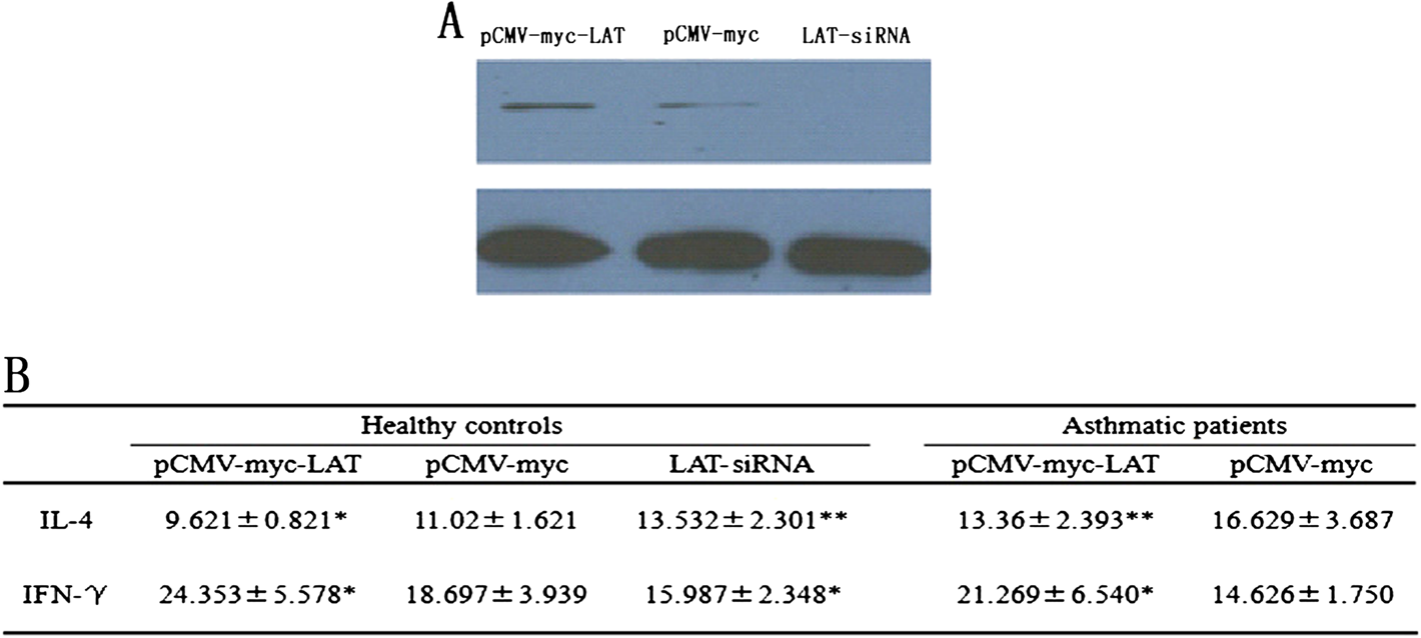
**Overexpression of LAT inhibits Th2 cytokine production and promoted Th1 cytokine production in human peripheral blood T cells in both asthmatic patients and healthy controls.** Purified peripheral blood T cells from allergic asthmatic patients and healthy controls were nucleofected with pCMV-myc-LAT, pCMV-myc, LAT-siRNA plasmids separately. **A**) LAT expression was analyzed by western blot. **B**) Nucleofected T cells were activated with anti-CD3 and anti-CD28 for 24 h. Cytokine production of IL-4 and IFN-γ was measured by ELISA. Data is shown as mean ± SD, n = 10 (*p < 0.05).

To clarify whether LAT can regulate human peripheral blood T cells differentiation in allergic asthma, Cytokine production was tested *in vitro*. After necleofection with pCMV-myc-LAT, pCMV-myc or LAT-siRNA plasmids into peripheral blood T cells, the expression of LAT protein was higher in allergic asthmatic patients compared to healthy controls (Figure 2A). Twenty-four hours stimulation of nucleofected T cells was used to investigate the production of IL-4 and IFN-γ, which representing Th2 and Th1 differentiation. IL-4 production was significantly reduced in peripheral blood T cells with overexpression of LAT (pCMV-myc-LAT) compared to control (pCMV-myc) in both healthy controls and asthmatic patients, while IFN-γ production was increased. Conversely, LAT deficient (LAT-siRNA) displayed increased IL-4 expression and decreased IFN-γ expression in healthy controls (Figure 2B). The results confirmed that LAT can be a crucial factor regulating human T cell differentiation *in vitro*.

### Histone hypoacetylation inhibits LAT transcription

**Figure 3:**
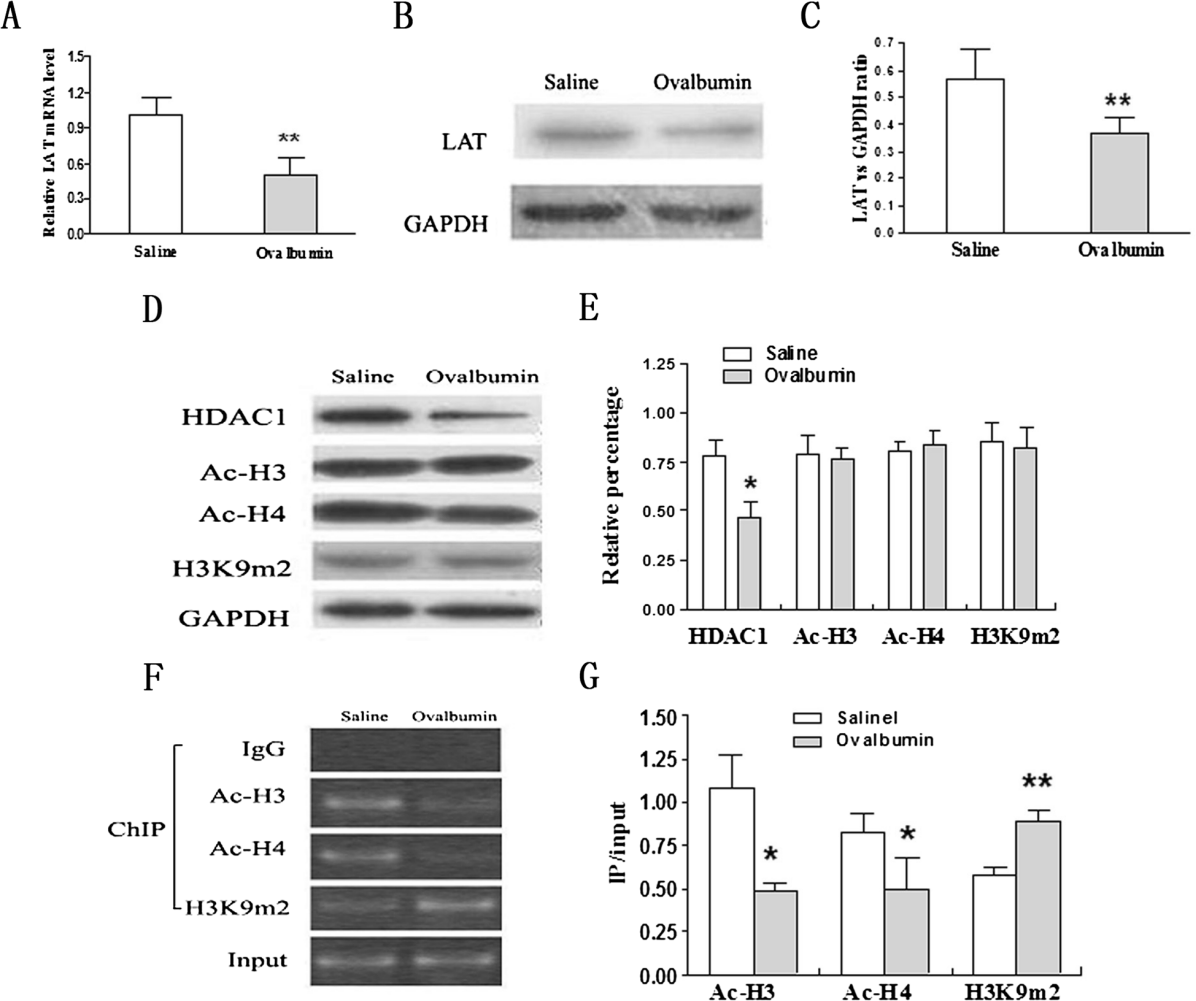
**Histone hypoacetylation inhibits LAT transcription in lung T cells after OVA exposure. A**) The mRNA expression of LAT was determined by RT-PCR in lung T cells isolated from OVA-immunized rats. Non-immunized rats were used as control. The mRNA expression of LAT was normalized to β-actin and shown as the relative content of LAT mRNA. **B**) and **C**) Immunoblot analysis of LAT expression in lung T cells isolated from immunized or non-immunized rats. Data is shown as LAT/GAPDH ratio. **D**) and **E**) Western blots analysis of the protein level of HDAC1, H3Ac, H4Ac and H3K9me2 in lung T cells isolated from immunized rats and non-immunized ones. GAPDH was used as a loading control. Data is shown as relative percentage. **F**) and **G**) ChIP assays of histone acetylation and methylation status on LAT promoter in lung T cells isolated from immunized rats and non-immunized ones. The data represents agarose gel electrophoretic separation of amplicons obtained after PCR (a representative experiment from a series of three is shown). Input DNA served as positive internal control for sample integrity. Data is shown as mean ± SD of triplicate samples. *p < 0.05; **p < 0.01; P-value were calculated from two or three independent experiments with consistent results.

To further determine the mechanism that T cell differentiation was regulated by LAT expression, an OVA immunized allergic airway inflammation rat model was applied. Consistent with the results in peripheral blood T cells from allergic asthmatic patients, both LAT mRNA (Figure 3A) and protein (Figure 3B-C) were greatly decreased in lung T cells in immunized rats compared to non-immunized ones.

To detect whether histone modifications were involved in regulating LAT expression in allergic asthma, the protein level of histones was measured in lung T cells in immunized rats and non-immunized ones. There was no significant different pattern in the expression of whole histone H3 and H4 acetylation and whole H3K9 dimethylation in lung T cells between immunized rats and non-immunized ones. However, the expression of HDAC1 was significantly decreased in lung T cells from immunized rats compared to non-immunized ones (Figure 3D-E).

To further illuminate whether histone modifications regulate LAT expression is directly or indirectly. Acetylation levels of histone H3 and H4 and dimethylation level of H3K9 at LAT upstream region was detected. CHIP analysis revealed that the acetylation level of histone H3 and H4 was markedly decreased and dimethylation level of H3K9 was dramatically increased in immunized rats compared to non-immunized ones (Figure 3F-G). These results indicate that lower expression of LAT in allergic airway inflammation may be regulated by decreased histone H3 and H4 acetylation and increased histone H3K9 dimethylation.

### TSA reverses the function of histone hypoacetylation and cytokine profiles in T cell differentiation *in vitro*

**Figure 4:**
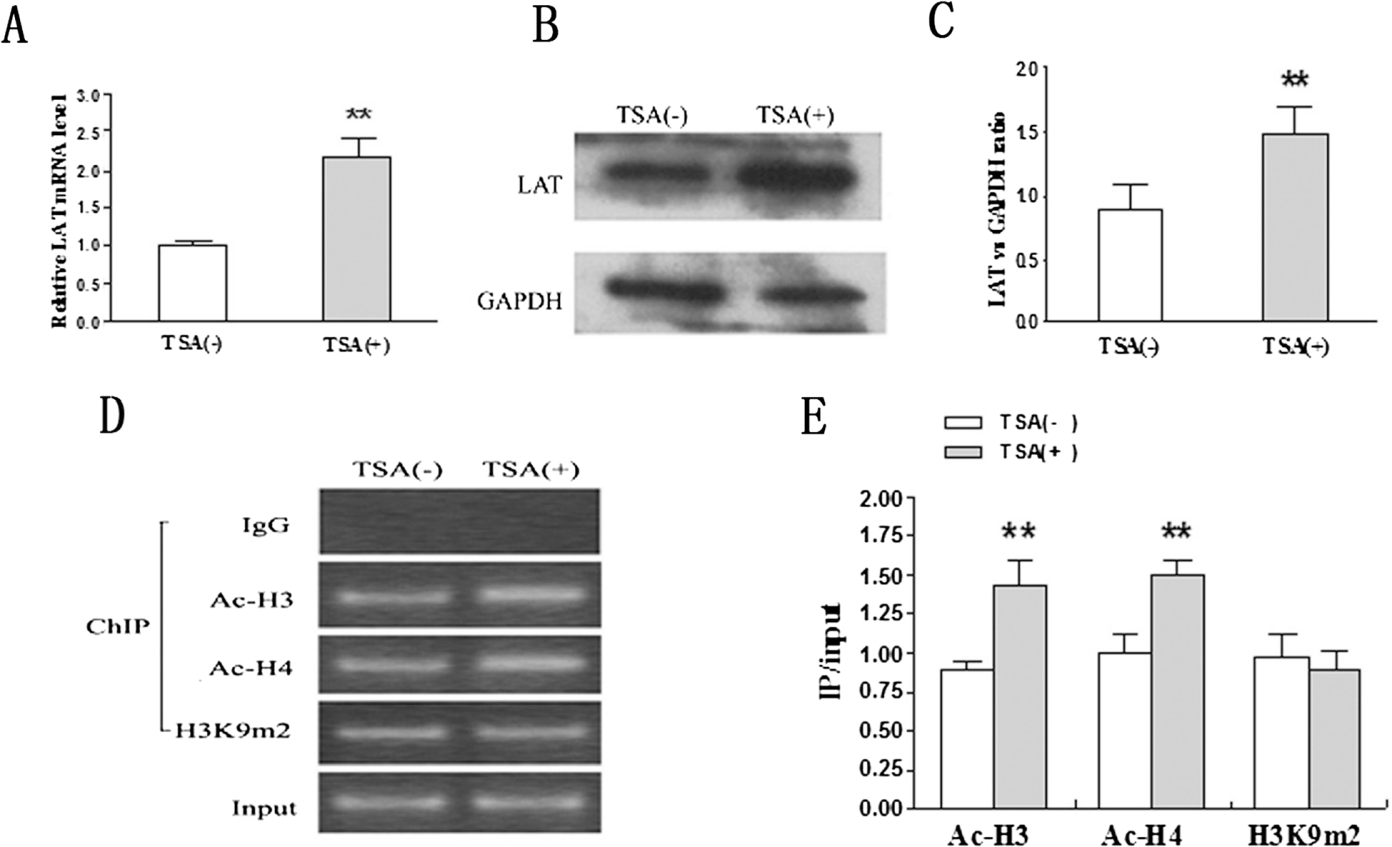
**TSA promotes LAT transcription and expression in lung T cells in OVA-immunized rats.** Lung T cells isolated from OVA-immunized rats were treated with or without TSA (2.5 ng/ml) for 24 h. **A**) The LAT mRNA level was determined by real-time PCR and normalized to β-actin. **B**)and **C**) LAT protein was determined by Western Blot and normalized to the housekeeping gene GAPDH. **D**) and **E**) ChIP assays of histone acetylation and methylation status on LAT promoter in lung T cells with or without TSA. The data represents agarose gel electrophoretic separation of amplicons obtained after PCR (a representative experiment from a series of three is shown). Input DNA served as positive internal control for sample integrity. Data is shown as mean ± SD, n = 10 (** p < 0.01).

Previous result showed that HDAC1 was largely reduced after OVA exposure, emphasizing the role of HDAC1 in allergic airway inflammation. TSA physically binds to HDAC molecules, which results in HDAC inhibition [21,22]. Therefore, it was hypothesized that TSA can regulate LAT transcription and expression by inhibiting HDACs. To determine it, lung T cells isolated from allergic airway inflammation rats were cultured with or without TSA for 24 hours. The level of LAT mRNA and protein were significantly induced by TSA (Figure 4A-B-C). To further determine whether TSA influences histone modifications, ChIP assay was performed. TSA markedly increased the level of acetylated histones H3 and H4 associated with the LAT upstream region in lung T cells (Figure 4D-E). However, there was no significant pattern in the dimethylation level of histone H3K9. Therefore, HDACs play important roles in repression of LAT transcription and expression, depending on LAT site-specific acetylation.

**Figure 5:**
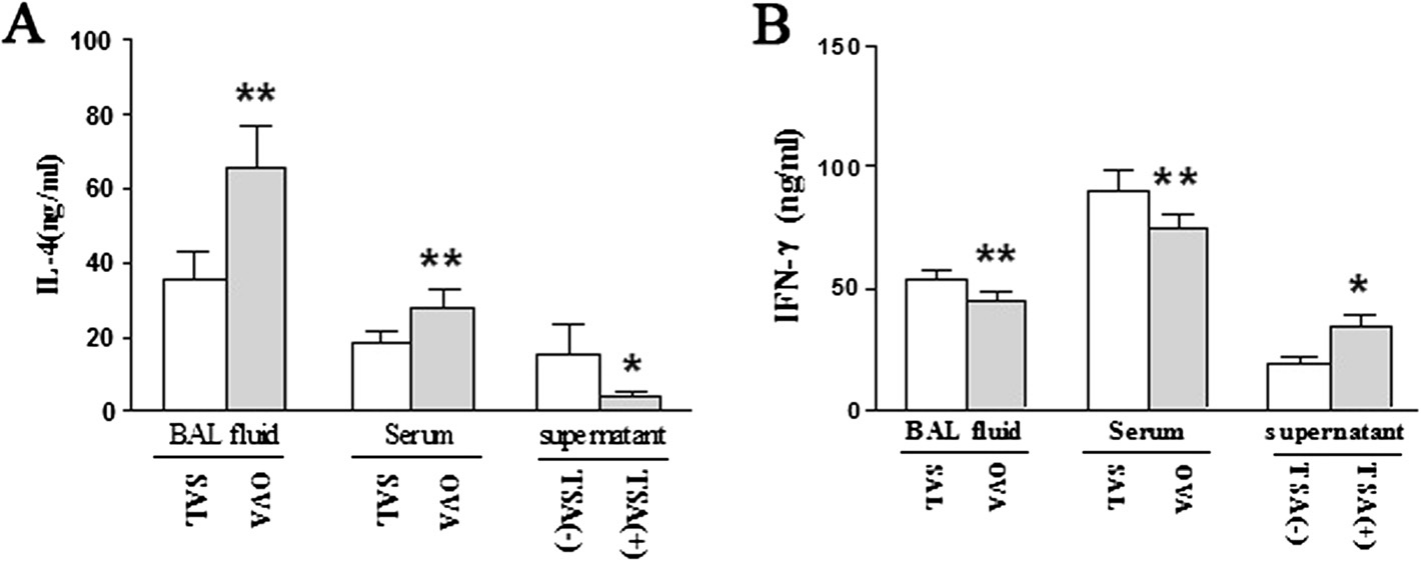
**TSA can reverse cytokine profiles in T cell differentiation *in vitro.*** BAL fluid and serum were harvested 24 hours after the last challenge. The concentration of IL-4 (**A**) and IFN-γ (**B**) in the BAL fluid and serum in OVA-immunized rats or non-immunized rats were measured directly by ELISA. Lung T cells isolated from OVA-immunized rats were cultured and IL-4 (**A**) and IFN-γ (**B**) in the supernatant were measured 24 h after incubation with or without TSA (2.5 ng/ml).

To rule out whether the cytokine profile can be reversed by treating lung T cells with TSA *in vitro*, expression of IL-4 and IFN-γ in BALF and serum and lung T cells were analyzed by ELISA. As expected, IL-4 expression was upregulated and IFN-γ expression was downregulated both in BALF and serum in immunized rats compared to non-immunized ones (Figure 5A-B). Interestingly, there were significantly decreased IL-4 production and increased IFN-γ production in the lung T cells from immunized rats cultured with TSA compared to TSA negative sample (Figure 5A-B). Thus, TSA can reverse lung Th1 or Th2 cytokine profiles in allergic airway inflammation.

## Discussion

In the present study, we show that LAT mRNA was decreased in peripheral blood T cells from allergic asthmatic patients, suggesting the involvement of LAT in T cell differentiation in allergic asthma. Overexpression of LAT by Nucleofection in peripheral blood T cells enhanced Th1 differentiation. Conversely, in the absence of LAT, Th2 differentiation was driven. Furthermore, allergic airway inflammation rat model revealed that histone hypoacetylation of LAT promoter could inhibit the expression of LAT and enhanced Th2 differentiation in lung tissue *in vitro*. In addition, TSA,a HDACs inhibitor, enforced acetylation of histones H3 and H4 which promoted LAT expression and inhibited Th2 cytokine production.

During the past decades, the Th1/Th2 imbalance has been well documented in the pathogenesis of allergic asthma [23,24]. Even though several other T helper cells have been reported recently, the Th2 cell is the main effector involved in the development of allergic asthma [25]. However, the initiation of T cell differentiation in the disease is not well understood. LAT, a transmembrane adapter protein, has been reported to be necessary for T cell development and function [5]. Experiments using LAT-deficient mice indicate that T cells in theses mice are hyperactivated and undergo a huge expansion, causing a fatal lymphoproliferative autoimmune disease [6,7]. A recent study also observed an abnormal pattern of expression and localization of LAT in lipid rafts after in vitro activation of lupus T cell [26]. In peripheral blood T cell of allergic asthmatic patients, we detected profoundly reduced expression of LAT mRNA, and Th2 cytokine production was conversely related to the expression of LAT. These results are consistent with recent reports that mice homozygous for a single tyrosine mutation in LAT develop a Th2 “autoimmune” lymphoproliferative disorder with excessive amounts of Th2 cytokines [27]. In-vivo allergen-induced airway inflammation study reported that overexpression of LAT prevented the development of airway inflammation with pronouncing reduction of inflammatory cells and IL-4 in BALF [28]. Combination with our results here confirmed that LAT is involved in allergic asthma by regulating the type-2 immune responses.

Single nucleotide polymorphisms (SNP), as the third generation of heredity markers, are widely used to study the mechanism of the susceptibility in human complex diseases, and the design of individualized treatment [29-31]. In the current study, we didn’t find the diversity of SNP in promoter, external and inter from peripheral blood T cells from allergic asthmatic patients (data not shown), suggesting that other factors may be involved in regulating LAT expression.

Histones are capable of being post-translationally modified by acetylation, methylation, ubiquitination or phosphorylation, all of which have been implicated in regulation of gene expression [32,33]. It was hypothesis that histone modifications can regulate LAT expression. As expected, it showed dramatically reduced histone H3 and H4 acetylation and significantly increased histone H3K9 dimethylation on LAT promoter in lung T cells from asthmatic rats. Therefore, histone modifications on LAT promoter may be gene-specific in lung T cells of allergic airway inflammation. Moreover, we found that the expression of HDAC1 in lung T cells was decreased in asthmatic rats, which is consistent with the report that the endogenous HDAC activity plays a pivotal role in preventing pre-established cytokine responses from deviating toward excessive Th2-like immunity [34]. Our data indicates that histone modifications may affect the development of type2 immune response by regulating LAT.

TSA is a reversible and specific HDAC inhibitor that increases histone acetylation and deregulates gene expression [21,22]. Previous study has shown that TSA attenuates the development of allergic airway inflammation by decreasing expression of the Th2 cytokines, which resulted from reduced T cell infiltration in the lung tissue [35]. By treating lung T cells with or without TSA *in vitro*, we observed that TSA markedly increased the level of histones H3 and H4 acetylation on LAT promoter in lung T cells. In addition, LAT gene transcription and protein expression were both increased by TSA in lung T cells isolated from allergic airway inflammation rats. Furthermore, TSA has the ability to reverse cytokine profiles by inhibiting Th2 cytokine production and enhancing Th1. The above data argues that HDACs have a crucial role in repression of the LAT expression in allergic airway inflammation.

## Conclusions

Thus, our study highlights the importance of LAT expression in allergic asthma and suggests that histone modification can influence LAT expression and regulate allergic airway inflammation. Our findings have important implications for therapeutically targeting LAT in allergic asthma.

## Competing interests

The authors confirm that there is no competing interest.

## Authors’ contributions

All authors read and approved the final manuscript. CYL and JP carried out the molecular genetic studies, participated in the sequence alignment and drafted the manuscript; LPR participated in the human study subjects; LXG and QL carried out the immunoassays and set up asthma model; XJL and WG performed the statistical analysis; XJG conceived of the study, and participated in its design and coordination and helped to draft the manuscript.
